# Emerging adulthood: prediction by markers of adulthood and associations with health in a Russian sample

**DOI:** 10.3389/fpubh.2025.1542170

**Published:** 2025-06-18

**Authors:** Oxana Mikhaylova

**Affiliations:** ^1^Department for Social Institutions Analysis, Faculty of Social Sciences, School of Sociology, HSE University, Moscow, Russia; ^2^Centre for Modern Childhood Research, Institute of Education, HSE University, Moscow, Russia

**Keywords:** emerging adulthood, markers of adulthood, inventory of the dimensions of emerging adulthood, Russia, health

## Abstract

**Introduction:**

Markers of the transition from adolescence to adulthood are changing in many countries, with traditional milestones such as marriage, parenthood, and financial independence becoming less central. This study investigates how changes in the emerging adulthood mindset relate to various maturity markers—including relationship status, parenthood, employment, education, material well-being, independent living—as well as subjective health indicators.

**Methods:**

Data were collected from two consecutive waves (2021 and 2022) of the Trajectories in Education and Careers (TrEC) longitudinal study, involving 2,663 Russian young adults aged 24 to 28 (54.5% female). Correlation, t-test, ANOVA, and regression analyses were used to assess associations between changes in IDEA (Inventory of the Dimensions of Emerging Adulthood) scores and changes in maturity markers and health indicators over a one-year period.

**Results:**

The analyses revealed that having children and higher material well-being were significantly associated with changes in the emerging adulthood mindset. In contrast, other traditional markers—such as relationship status, education, and living with parents—were not significantly associated. Several health indicators, including subjective health status, healthy lifestyle, prior mental health specialist consultation, and anxiety, were also linked to changes in the emerging adulthood mindset.

**Discussion:**

These findings indicate that not all traditional markers of adulthood are relevant for emerging adults today. The results highlight the growing importance of health-related factors in understanding the transition to adulthood, suggesting a shift in the criteria that define this life stage.

## Introduction

In many developed countries, the boundary between adolescence and adulthood has become increasingly blurred (1), perhaps because traditional rites of passage-such as getting married and having children-have become less central. Authors of a recent meta-analysis of studies using markers of maturity scales (2) suggest that not all characteristics of adulthood are seen as aspects of emerging adults’ identities. Emerging adulthood is defined as a distinct developmental period characterized by instability, self-focus, feeling “in-between” adolescence and full adulthood, identity exploration, and a sense of possibilities for the future (3). The Inventory of Dimensions of Emerging Adulthood (IDEA) (4) is a measurement tool designed to assess this emerging adult mindset by capturing whether individuals perceive this life stage as a time for experimentation, instability/negativity, other-focus, self-focus, and feeling in-between.

### Cultural specificity of emerging adulthood

It may be difficult to make generalizations on this issue, however, as markers of adulthood differ between countries, perhaps due to variation in factors such as social inequality, material well-being, gender equality, and ethnic diversity. For example, individuals from Western, individualistic societies such as the United States are more likely to view personal responsibility and independent decision-making as key indicators of adulthood (2). In contrast, people from more collectivist cultures, such as China or India, tend to emphasize traditional milestones like marriage, parenthood, and fulfilling family obligations as central to attaining adult status (2). As Arnett (3) has noted, markers of adulthood might reflect maturation for those in some countries but be obsolete in others.

### Markers of adulthood in Russia

Although the theory of EA was originally developed in the United States, research in Russia has shown that many young adults also identify with EA features such as identity exploration, experimentation, and self-focus (5). However, the extent to which individuals experience this life stage is shaped by local social, economic, and cultural factors. In Russia, delayed transitions to marriage and parenthood, rising participation in higher education, and significant socioeconomic disparities influence both the timing and meaning of adulthood transitions (5). For example, young people from higher socioeconomic backgrounds or with higher education are more likely to perceive their current life stage as a time of opportunity and self-exploration, while those with fewer resources tend to assume adult roles earlier.

Additionally, regional and cultural diversity within Russia further shapes how emerging adulthood is experienced, with traditional norms and economic constraints limiting the possibilities for prolonged identity exploration for some groups (5). First, income varies substantially around the country. According to Rosstat (6), the monthly average income in Russia’s central area in 2022 (83,126 rubles) was higher than the national average (65,338 rubles), which was twice that of the North Caucasian Federal District (37,361 rubles). Second, although women are active in a variety of economic sectors in Russia, their incomes are typically lower than those of men, their involvement in humanitarian causes is greater than that of men, and they are less likely to be employed in STEM fields (7). Third, Russia is ethnically and culturally diverse (8). Fourth, it has a heterogeneous healthcare infrastructure (9), which means that some Russian regions provide better access to healthcare than others. Understanding these cultural differences is essential for interpreting the applicability and meaning of emerging adulthood in Russia.

In line with Norman et al. (10), the revised set of adulthood markers used in this study includes relationship status (married or in a stable romantic relationship), education status (currently studying or not), level of material well-being, living arrangement (living with parents or independently), parental status (having children or not), employment status, and substance use behaviors (frequency of heavy alcohol consumption and cigarette smoking), each reflecting key domains reliably linked to the transition to adulthood. These markers collectively constitute the subscales of the adulthood criteria used in the present analysis.

### Health and emerging adulthood

The rationale for examining both markers of adulthood and IDEA in relation to health indicators stems from growing evidence that the transition to adulthood significantly impacts both mental and physical well-being (11, 12). Research has demonstrated that perceptions of an unsuccessful transition to adulthood-characterized by negativity and instability-positively predict stress and somatic physical health concerns, while positive transition dimensions (experimentation/possibilities) negatively predict these outcomes (11). Similarly, Baggio et al. (13) found that emerging adulthood is a period of significant changes in psychosocial well-being, with important implications for health behaviors and outcomes.

The inclusion of both mental and physical health indicators in this study is further justified by research showing that substance use behaviors, which are often established during emerging adulthood, are associated with dimensions of the IDEA (14). Additionally, psychological indicators have been identified as significant markers of a successful transition to adulthood, encompassing aspects such as emotional wellness, maintenance of healthy relationships, and constructive engagement in educational and occupational activities (15).

The pandemic, economic shocks, and other recent events in Russia have likely impacted the development of health services, particularly those related to mental health (16, 17). Psychological state can influence both markers of adulthood and IDEA items, especially those reflecting relative maturity and independence (15). Research shows that emerging adults who feel they are falling behind in milestones like employment or relationships often report higher stress, anxiety, and depression, with this relationship being bidirectional (18, 19). Empirical studies confirm that unmet expectations regarding adulthood markers are linked to poorer mental health (20), and those who have not achieved these markers experience higher stress and lower well-being (21). Additionally, emotional and general health, partnership, and employment are strong predictors of well-being during this transition (22), while gender and socioeconomic status further shape these associations (23).

By examining both objective markers of adulthood and subjective perceptions of emerging adulthood in relation to health indicators, this study aims to provide a comprehensive understanding of how the transition to adulthood relates to well-being in contemporary Russian young adults, contributing to the broader literature on emerging adulthood across diverse cultural contexts.

Despite growing interest in the applicability of emerging adulthood theory in non-Western contexts, it remains unclear which markers of adulthood are most relevant for Russian young adults and how these markers, along with mental and physical health indicators, are associated with changes in the emerging adulthood mindset. This study addresses this gap by systematically examining the relationships between revised markers of adulthood, health indicators, and changes in emerging adulthood mindset in a nationally representative Russian sample.

### Aims and hypotheses

The present study aims to examine longitudinal, predictive relationships between specific markers of adulthood, subjective health indicators, and changes in the emerging adulthood (EA) mindset over a one-year period among Russian young adults. Using data from two annual waves of the TrEC longitudinal study (2021 and 2022), I assess which markers of adulthood and health indicators are significant predictors of positive or negative changes in IDEA scores, which operationalize the EA mindset.

Hypotheses:

- H1 (Predictive): I hypothesize that achieving greater maturity-as indicated by certain markers of adulthood (relationship status, parenthood, employment, education status, material well-being, living arrangement, and substance use behaviors)-will be associated with a decrease in IDEA scores over one year (i.e., a less pronounced emerging adulthood mindset). I do not expect all markers to have equal effects; rather, we anticipate that some markers (especially parenthood and material well-being, based on prior research and preliminary findings) will show stronger associations than others.
- H2 (Predictive): I hypothesize that positive changes in subjective health indicators (e.g., better self-rated health, healthy lifestyle, lower anxiety, and prior mental health specialist consultation) will be associated with increases in IDEA scores over one year, indicating a stronger emerging adulthood mindset among those who perceive their health more favorably.

In this paper, “emerging adulthood (EA) mindset” is operationalized by the IDEA scale; higher IDEA scores indicate a stronger EA mindset, while lower scores indicate a weaker EA mindset. Thus, a decrease in EA mindset refers to a reduction in IDEA scores, and an increase refers to higher IDEA scores.

## Method

### Procedure

The data for this research come from the Trajectories in Education and Careers (TrEC), a longitudinal study tracking Russian youth from school to adulthood, which was initiated in 2009 (24). The TrEC’s initial sample had an average age of 14 years (25). Survey questions in the study are changed slightly each year, though the section related to education largely stays the same (25). The study was not preregistered.

### Participants

The present study uses data from two recent waves of the TrEC, the 2021 (*N* = 2,981) and 2022 (*N* = 2,663) surveys. Our sample comprises 2,663 Russian citizens aged 24 to 28 (Mbirth year = 1996, SD = 0.5). The sample was 54.5% female, with 79.1% of participants identifying as Russian and 57.4% having completed higher education. This age range is generally considered late emerging adulthood, illustrating the trend of delayed emerging adulthood noted by academics in the field (26).

### Measures

All survey questions can be found online through the following link: https://trec.hse.ru/data/2023/03/06/2035844255/%D0%B0%D0%BD%D0%BA%D0%B5%D1%82%D0%B011.pdf.

### Markers of adulthood

#### Role transitions

Measures assessing changing life roles comprised being in a relationship or married, having at least one child, and ongoing involvement in education (yes or no). Compared to 2021, participants in 2022 were less likely to be in romantic relationships and reside with their parents (Supplementary Table 1). The proportion of participants currently pursuing education and those who were childless decreased over the period.

#### Independence

Level of independence was measured by subjective family material well-being [for more details, see, for instance, this paper (5)], whether the respondent lived with their parents, and whether they were employed.

#### Norm compliance

Two indicators of substance use were included as measures of norm compliance: alcohol consumption, defined as drinking at least once per week (yes/no), and smoking, defined as using tobacco products more than once per week (yes/no).

### IDEA

The brief version of the IDEA was used in the study to gauge attitudes regarding emerging adulthood (Supplementary Table 2). The scale featured four items about identity exploration, experimentation and possibilities, and self-focus. Cronbach’s alpha was 0.809 in 2021 and 0.816 in 2022, indicating that internal consistency was high. Total scores ranged from 4 to 16 across the two time points with means of 12.11 (2.67) in 2021 and 11.8 (2.67) in 2022. This was a statistically significant difference, indicating that participants were less likely to define their experiences as those of emerging adults in 2022. The EA mindset was operationalized using the IDEA scale, where higher scores reflect a stronger identification with the characteristics of emerging adulthood.

### Mental and physical health

Participants’ mental health was gauged by items asking if they had ever sought help from a mental health professional, how often they experienced stress, and their level of anxiety over the past year. Level of anxiety about the future was also assessed (Supplementary Table 3). Physical health was assessed by participants’ subjective assessment of their health and whether they led a healthy lifestyle. All health measures were only recorded in 2022.

### Demographics

Demographic data collected included age, gender, higher education status (yes or no), and nationality (Russian or non-Russian) (Supplementary Table 4). Gender was measured as a binary variable as it is currently against the law to discuss non-binary gender in Russia (27). Previous research using the IDEA has shown inconsistent associations with demographic variables (28, 29). For example, some studies have found that gender and age are linked to certain IDEA subscales, while others report no significant associations or conflicting patterns across cultural contexts (28–30).

### Data analysis

All analyses were conducted using R Studio. To examine overall changes in emerging adulthood mindset between 2021 and 2022 within the same participants, I first conducted paired-sample t-tests on IDEA scores. This test was chosen because it is appropriate for assessing mean differences across two time points in a longitudinal design with repeated measures. Where relevant, I also considered repeated-measures ANOVAs to assess within-subject changes for variables with more than two levels or to test for interaction effects.

To explore associations between study variables and IDEA scores, I used independent-samples t-tests, one-way ANOVAs, and Pearson correlation coefficients, depending on the measurement level and distribution of each variable. These tests allowed me to compare mean IDEA scores across groups (e.g., by relationship status, employment, parenthood) and to assess linear associations with continuous predictors (e.g., material well-being, anxiety).

For explanatory analysis, I conducted linear regression to identify which variables predicted changes in IDEA scores over the one-year period. Variables included in the regression were selected based on having the most substantial effect sizes (Cohen’s d > 0.20) in preliminary group comparisons, ensuring that only predictors showing meaningful differences were retained. Specifically, the regression included having children, subjective level of material well-being, and whether an individual had ever seen a mental health specialist. Subjective health evaluation was not included in the regression due to high collinearity with mental health specialist use, which would have introduced multicollinearity.

This analytic strategy allowed me to (1) confirm whether significant overall change in IDEA scores occurred, (2) identify which demographic, adulthood marker, and health variables were associated with these changes, and (3) determine which factors most strongly predicted longitudinal change in the emerging adulthood mindset.

## Results

### Descriptive statistics and correlations

Supplementary Tables 5–7 show the results of t-tests, ANOVAs, and correlations between variables. Correlation analysis showed that the IDEA total score was positively associated with completion of higher education, employment, having children, material well-being, having a healthy lifestyle, subjective health status, and previous experience seeing a mental health professional.

Regarding changes in IDEA scores from 2021 to 2022, smaller decreases were found in young people who had completed higher education compared to those who had not, and in those who were employed compared to unemployed participants. Participants that reported greater material well-being had small positive changes in IDEA scores. Participants who had children showed bigger decreases in IDEA scores than those who did not. Those who had seen a mental health specialist showed bigger increases in IDEA scores. Those who reported not having a healthy lifestyle had a bigger decrease in IDEA scores. Regarding changes in anxiety, the larger the increase, the lower the IDEA score.

### Regression analysis

A regression model was fitted to predict changes in IDEA scores from 2021 to 2022 (Supplementary Table 8). A positive difference between IDEA scores in 2022 and 2021 indicates an increase in the emerging adulthood mindset, while a negative difference reflects a decrease. Three indicators-material well-being, having children, and previous experience seeing a mental health specialist-were added as predictors. These variables were selected because, among all potential predictors in Supplementary Table 1 (excluding the IDEA scale), they showed the largest effect sizes, with Cohen’s d values above 0.20 when comparing group means between 2021 and 2022. Cohen’s d was calculated as the standardized mean difference between the two years for each variable, providing a measure of practical significance for observed changes (rather than statistical significance alone). This threshold was used to focus the regression analysis on variables with the most substantial year-to-year changes, as indicated by their effect sizes from independent samples t-tests.

Results indicated that these three predictors explained 4.6% of the variance in changes in IDEA scores, *R*^2^ = 0.05, F(3,2,115) = 33.69, *p* < 0.001. Having children was associated with a significant decrease in IDEA scores, b = −1.24, 95% CI [−1.60,−0.89], *p* < 0.01, while higher subjective material well-being (b = 0.48, 95% CI [0.32,0.63], p < 0.01) and previous experience seeing a mental health specialist (b = 0.67, 95% CI [0.28,1.06], p < 0.01) were associated with increases in IDEA scores.

## Discussion

The aim of this study was to assess predictors of changes in an emerging adulthood (EA) mindset. My findings show that not all markers of adulthood are related to such changes. Results revealed that living with parents, currently being in education, relationship status, and smoking and drinking frequency were not associated with changes in an EA mindset assessed with the IDEA scale. This partly supports our first hypothesis.

It is possible that living with one’s parents is not connected with an EA mindset because psychological separation from one’s caregivers is more important than physical separation in feeling like an emerging adult. Furthermore, it may be difficult for young people to gain physical separation from their parents due to the high price of rented accommodation. Regarding being in education, it may be that this was not connected to EA mindset change in the present sample as many of those still in education also worked. Being in a relationship was also unrelated to feeling like an emerging adult, perhaps due to its negating effect on self-focus.

Smoking and drinking frequency were not related to change in EA mindset in this sample. Previous research on substance use during emerging adulthood has produced inconsistent findings: some studies report that risky behaviors such as alcohol use and smoking are associated with lower psychosocial maturity or a less pronounced emerging adulthood mindset, while others find weak or no associations, or that these relationships vary by age, gender, or context (26, 31, 32). For example, some work suggests that substance use peaks in the early twenties and then declines as individuals take on more adult roles (33, 34), but other studies show that college attendance, gender, and cultural factors can moderate these trends, leading to different patterns across samples (31, 32). In line with these mixed results, the present study found no significant association between smoking or drinking frequency and changes in the emerging adulthood mindset (26, 31, 32).

A number of health-related variables, assessed only at time 2, were associated with changes in EA mindset. These included whether participants had previously seen a mental health specialist, whether they had a healthy lifestyle, their subjective health status, and reported changes in anxiety over the previous year. These findings correspond with studies from other countries which show that health is an important correlate of an emerging adult mindset (11, 13, 35). It is possible that those who feel younger score higher on markers of health and are more likely to engage in self-care.

None of the sociodemographic variables measured in this study were associated with changes in EA mindset. This may reflect that in the small age range assessed here and in Russia specifically, there is comparable likelihood of feeling like an emerging adult, regardless of demographic factors. Also, improvements in gender equality have led to the greater involvement of women in the labor market, diminishing possible gender differences (36). Regarding education, the recent trend for lifelong education may have resulted in a lack of significant association with EA mindset (37, 38). Likewise, nationality was not related to IDEA score changes, perhaps because of Russia’s attempts to integrate those of different nations and ethnicities (39).

### Limitations and future directions

A number of limitations may affect the interpretation of these findings. First, the study did not include all items from the IDEA full scale. As such, it was not possible to investigate how other aspects of the IDEA, such as negative emotions and other-focus, could be explained by the selected predictors. Future studies could be conducted with this in mind. Second, the regression model explained less than 10% of the variation in changes in IDEA scores, indicating that consideration of other variables is needed to better predict changes in the emerging adulthood mindset. Third, data were solely quantitative, meaning a qualitative approach could be the focus of future studies. This could help to assess participants’ understanding of items in the IDEA scale. Participants who give the same answers may have different beliefs or interpretations of the items, as some studies using the IDEA have shown previously (40–42). Additionally, it should be noted that while items from the TrEC were matched to the MoA subscales of role transitions, independence, and norm compliance, there were no matches for the relative maturity subscale due to the absence of comparable items in the TrEC dataset. As a result, the study could not assess the role of relative maturity as a marker of adulthood, which may limit the comprehensiveness of the findings regarding the full spectrum of adulthood markers. Furthermore, no physical health markers were included in the regression analyses, so this study focused primarily on self-reported mental health indicators. This could be an area for future research, and future studies should consider including or developing measures that capture both relative maturity and physical health dimensions.

## Conclusion

This study investigated associations between changes in IDEA scores over time and markers of maturity and subjective health in Russians aged 24–26. All health indicators were shown to be significantly correlated with changes in IDEA scores. Because not all markers of maturity were associated with IDEA scores, findings highlight the importance of updating and reconsidering markers of adulthood, in line with recent academic discussions.

## Data Availability

The original contributions presented in the study are included in the article/Supplementary material, further inquiries can be directed to the corresponding author.

## References

[1] Sharon T. Constructing adulthood: markers of adulthood and well-being among emerging adults. Emerg Adulthood. (2016) 4:161–7. doi: 10.1177/2167696815579826

[2] Wright M Oxley F Von Stumm S. Measuring adulthood – a Meta-analysis of the markers of adulthood scale. Eur J Psychol Assess. (2024) 40:515–28. doi: 10.1027/1015-5759/a000873

[3] Arnett J. Conceptions of the transition to adulthood: perspectives from adolescence through midlife. J Adult Dev. (2001) 8:133–43. doi: 10.1023/A:1026450103225

[4] Reifman A Arnett JJ Colwell MJ. Emerging adulthood: theory, assessment and application. J Youth Dev. (2007) 2:37–48. doi: 10.5195/jyd.2007.359

[5] Mikhaylova O Sivak E. Emerging adulthood in Russia: class and educational disparities. Emerg Adulthood. (2023) 11:1346–55. doi: 10.1177/21676968231206208, PMID: 40297624

[6] Rosstat. The labour market, employment and wage. (2023). Available online at: https://rosstat.gov.ru/labor_market_employment_salaries [Accessed August 30, 2023]

[7] Rebrey SM. Gender inequality in Russia: axial institutions and agency. Russian J Econ. (2023) 9:71–92. doi: 10.32609/j.ruje.9.94459

[8] Bufetova AN Khrzhanovskaya AA Kolomak EA. Cultural heterogeneity and economic development in Russia. J Siberian Fed Univ Human Soc Sci. (2020) 13:453–63. doi: 10.17516/1997-1370-0582

[9] Shartova N Tikunov V Chereshnya O. Health disparities in Russia at the regional and global scales. Int J Equity Health. (2021) 20:1–16. doi: 10.1186/s12939-021-01502-6, PMID: 34256759 PMC8276545

[10] Norman KB Grahe JE Lee S. Reconstructing adulthood: revising the markers of adulthood scale for increased ecological validity. Psychol Rep. (2023) 126:1042–61. doi: 10.1177/00332941211061700, PMID: 34894879

[11] Barlett CP Barlett ND Chalk HM. Transitioning through emerging adulthood and physical health implications. Emerg Adulthood. (2020) 8:297–305. doi: 10.1177/2167696818814642

[12] Lisha NE Grana R Sun P Rohrbach L Spruijt-Metz D Reifman A. Evaluation of the psychometric properties of the revised inventory of the dimensions of emerging adulthood (IDEA-R) in a sample of continuation high school students. Eval Health Prof. (2014) 37:156–77. doi: 10.1177/0163278712452664, PMID: 22786874 PMC3796175

[13] Baggio S Studer J Iglesias K Daeppen J-B Gmel G. Emerging adulthood: a time of changes in psychosocial well-being. Eval Health Prof. (2017) 40:383–400. doi: 10.1177/0163278716663602, PMID: 27573914

[14] Allem J-P Sussman S Unger JB. The revised inventory of the dimensions of emerging adulthood (IDEA-R) and substance use among college students. Eval Health Prof. (2017) 40:401–8. doi: 10.1177/0163278716660742, PMID: 27468859 PMC5550352

[15] Tavakkoli M Valarezo E García LF. Perceptions of adulthood and mental health. IJERPH. (2024) 21:773. doi: 10.3390/ijerph21060773, PMID: 38929019 PMC11204109

[16] Borozdina E Zvonareva O. Medical professionals’ agency and pharmaceuticalization: physician-industry relations in Russia. Health (London). (2024) 28:108–25. doi: 10.1177/13634593221116508, PMID: 35913030 PMC10714710

[17] Jurcik T Jarvis GE Zeleskov Doric J Krasavtseva Y Yaltonskaya A Ogiwara K. Adapting mental health services to the COVID-19 pandemic: reflections from professionals in four countries. Couns Psychol Q. (2021) 34:649–75. doi: 10.1080/09515070.2020.1785846

[18] Brito AD Soares AB. Well-being, character strengths, and depression in emerging adults. Front Psychol. (2023) 14. doi: 10.3389/fpsyg.2023.1238105, PMID: 37809290 PMC10552671

[19] Mund M Neyer FJ. The winding paths of the lonesome cowboy: evidence for mutual influences between personality, subjective health, and loneliness. J Pers. (2016) 84:646–57. doi: 10.1111/jopy.12188, PMID: 26112403

[20] Culatta E Clay-Warner J. Falling behind and feeling bad: unmet expectations and mental health during the transition to adulthood. Soc Mental Health. (2021) 11:251–65. doi: 10.1177/2156869321991892

[21] Bao G Xie H Cheng X Wang C Hong J. Adulthood status and its associations with stress and well-being among Chinese emerging adults: a person-centred approach. Int J Psychol. (2023) 58:605–13. doi: 10.1002/ijop.12936, PMID: 37661857

[22] Wang RAH Haworth C. Life-course predictors of wellbeing in emerging adulthood. (2020).

[23] Matud MP Ibáñez I Hernández-Lorenzo DE Bethencourt JM. Gender, life events, and mental well-being in emerging adulthood. Int J Soc Psychiatry. (2023) 69:1432–43. doi: 10.1177/00207640231164012, PMID: 37029493

[24] HSE University. Trajectories in education and careers (TrEC). (2022). Available online at: https://trec.hse.ru/en/ [Accessed March 24, 2022]

[25] Malik V. The Russian panel study ‘trajectories in education and careers.’. Longit Life Course Stud. (2019) 10:125–44. doi: 10.1332/175795919X15468755933416

[26] Nelson LJ. The theory of emerging adulthood 20 years later: a look at where it has taken us, what we know now, and where we need to go. Emerg Adulthood. (2021) 9:179–88. doi: 10.1177/2167696820950884

[27] Consultant. Article 6.21. Propaganda of non-traditional sexual relations and/or preferences, gender change, refusal of childbearing. (2025). Available online at: https://www.consultant.ru/document/cons_doc_LAW_34661/5b103e878283a04f8ea2130f38e19f10b516b626/ [Accessed May 1, 2025]

[28] Kuang J Zhong J Yang P Bai X Liang Y Cheval B. Psychometric evaluation of the inventory of dimensions of emerging adulthood (IDEA) in China. Int J Clin Health Psychol. (2023) 23:100331. doi: 10.1016/j.ijchp.2022.100331, PMID: 36247406 PMC9529670

[29] Zorotovich J Johnson EI. Five dimensions of emerging adulthood: a comparison between college students, nonstudents, and graduates. Coll Stud J. (2019) 53:376–84.

[30] Sánchez-Queija I Parra Á Camacho C Arnett J. Spanish version of the inventory of the dimensions of emerging adulthood (IDEA-S). Emerg Adulthood. (2020) 8:237–44. doi: 10.1177/2167696818804938

[31] Baggio S Iglesias K Studer J Gmel G. An 8-item short form of the inventory of dimensions of emerging adulthood (IDEA) among young Swiss men. Eval Health Prof. (2015) 38:246–54. doi: 10.1177/0163278714540681, PMID: 24973242

[32] Zhu JY Goldstein AL Mackinnon SP Stewart SH. Alcohol use and emerging adult development: a latent profile analysis of community drinkers. Int J Ment Health Addiction. (2019) 17:1180–99. doi: 10.1007/s11469-018-0039-x

[33] Arnett JJ. The developmental context of substance use in emerging adulthood. J Drug Issues. (2005) 35:235–54. doi: 10.1177/002204260503500202

[34] Gates JR Corbin WR Fromme K. Emerging adult identity development, alcohol use, and alcohol-related problems during the transition out of college. Psychol Addict Behav. (2016) 30:345–55. doi: 10.1037/adb0000179, PMID: 27077443 PMC4877261

[35] McCoy SS Chou CP Grahe JE Miller TJ Photias KL. A comparative study of four-year and community college students’ subjective experiences of emerging adulthood, belonging needs, and well-being. Routledge Open Res. (2023) 1:17. doi: 10.12688/routledgeopenres.17573.2

[36] Kay R. A liberation from emancipation? Changing discourses on women’s employment in soviet and post-soviet Russia In: Russia After Communism: Routledge (2020). 51–72.

[37] Korshunov I Gaponova O Peshkova V. Live and learn: continuous education in Russia. Moskov: Publishing House of Higher School of Economics. (2019). 22–23.

[38] Kapeliushnikov RI. Returns to education in Russia: nowhere below? Econ Quest. (2021):37–68. doi: 10.32609/0042-8736-2021-8-37-68

[39] Ryazantsev S. Modern migration policy of Russia: challenges and approaches to improvement. Sotsiologicheskie issledovaniya. (2019):117–26. doi: 10.31857/S013216250006666-5

[40] Côté JE. The dangerous myth of emerging adulthood: an evidence-based critique of a flawed developmental theory. Appl Dev Sci. (2014) 18:177–88. doi: 10.1080/10888691.2014.954451

[41] Landberg M Lee B Noack P. What alters the experience of emerging adulthood? How the experience of emerging adulthood differs according to socioeconomic status and critical life events. Emerg Adulthood. (2019) 7:208–22. doi: 10.1177/2167696819831793

[42] Macek P Bejček J Vaníčková J. Contemporary Czech emerging adults: generation growing up in the period of social changes. J Adolesc Res. (2007) 22:444–75. doi: 10.1177/0743558407305417

