# Supplementary material for: Emerging adulthood: prediction by markers of adulthood and associations with health in a Russian sample
Source: Front Public Health. 2025 Jun 18;13:1542170. doi: 10.3389/fpubh.2025.1542170 (PMC12213429; doi:10.3389/fpubh.2025.1542170)
Supplement: Supplementary file 1 [file Supplementary_file_1.docx]

# Appendix

Table 1 presents descriptive statistics for primary markers of adulthood, including relationship status, education, material well-being, living arrangement, parenthood, employment, and substance use (alcohol and smoking). For categorical variables, percentages for each group are shown; for continuous variables, means and standard deviations (M, SD) are reported. Data are presented for both 2021 and 2022, along with effect sizes (Cohen’s d) for year-to-year changes. Higher Cohen’s d values indicate larger differences between years.

**Table 1**

Descriptive Statistics for Markers of Adulthood (2021 and 2022)

| Variable | 2021 % or M (SD) | 2022 % or M (SD) | Effect Size  (Cohen’s d) |
| --- | --- | --- | --- |
| Relationship status |  |  |  |
| Not in relationship | 36% | 53.5% |  |
| In relationship | 64% | 46.5% | -0.35 |
| Education status |  |  |  |
| Not studying | 81% | 88.2% |  |
| Studying | 19% | 11.8% | 0.19 |
| Material well-being (1–6) | 3.94 (1.05) | 3.8 (1.01) | 0.13 |
| Living with parents |  |  |  |
| Does not live with parents | 43.26% | 80.7% |  |
| Lives with parents | 36.74% | 19.3% | -0.38 |
| Parenthood |  |  |  |
| No children | 77.5% | 73.1% |  |
| At least one child | 22.5% | 26.9% | -0.10 |
| Employment |  |  |  |
| Unemployed | 22.4% | 24.6% |  |
| Employed | 77.6% | 75.4% | -0.05 |
| Heavy alcohol (weekly+) | – | 9.1% (yes) | – |
|  |  | 98.9% (no) |  |
| Smoking (≥ pack/week) | – | 14.5% (yes) | – |
|  |  | 85.5% (no) |  |

Table 2 displays descriptive statistics for the Inventory of the Dimensions of Emerging Adulthood (IDEA) total score and its subscales, including means and standard deviations for 2021 and 2022, as well as effect sizes (Cohen’s d). The IDEA scale measures the extent to which participants identify with emerging adulthood characteristics, with higher scores indicating a stronger emerging adulthood mindset.

**Table 2**

Descriptive Statistics for Emerging Adulthood Mindset (IDEA)

| Variable | 2021 M (SD) | 2022 M (SD) | Effect Size (Cohen’s d) |
| --- | --- | --- | --- |
| IDEA total (1–16) | 12.11 (2.67) | 11.8 (2.60) | 0.12 |
| Time for possibilities (1–4) | 3.02 (0.81) | 2.96 (0.80) | 0.07 |
| Time for experimentation, self/world exploration (1–4) | 3.11 (0.80) | 3.01 (0.79) | 0.12 |
| Time for individual freedom (1–4) | 2.87 (0.90) | 2.79 (0.91) | 0.09 |
| Time for self-analysis (1–4) | 3.04 (0.82) | 2.97 (0.80) | 0.08 |

Table 3 summarizes mental and physical health indicators assessed in 2022, including mental health specialist consultation, subjective health evaluation, healthy lifestyle, stress frequency, anxiety about the future, and changes in anxiety. For categorical responses, percentages for each option are provided; for continuous responses, means and standard deviations are shown.

**Table 3**

Descriptive Statistics for Mental and Physical Health Indicators (2022)

| Variable | 2022 % or M (SD) |
| --- | --- |
| Ever seen mental health specialist | 18% (yes), 82% (no) |
| Subjective health evaluation |  |
| Very good | 8.9% |
| Good | 49.6% |
| Ok | 35.2% |
| Bad | 2.6% |
| Very bad | 0.4% |
| Stress frequency |  |
| Never | 5.6% |
| Rarely | 27.8% |
| Sometimes | 36.8% |
| Frequently | 22.2% |
| Always | 7.5% |
| Anxiety about the future (1–10) | 4.42 (2.7) |
| Change in anxiety |  |
| Significantly decreased | 6.8% |
| Slightly decreased | 9.9% |
| Did not change | 28.1% |
| Slightly increased | 28.6% |
| Significantly increased | 26.6% |
| Declares healthy lifestyle | 54.4% (yes), 35.6% (no) |

Table 4 reports descriptive statistics for sociodemographic variables, including gender, year of birth, higher education status, and nationality, for both 2021 and 2022. Where applicable, effect sizes (Cohen’s d) for changes across years are also provided.

**Table 4**

Descriptive Statistics for Sociodemographic Variables (2021 and 2022)

| Variable | 2021 % or M (SD) | 2022 % or M (SD) | Effect Size  (Cohen’s d) |
| --- | --- | --- | --- |
| Gender |  |  |  |
| Male | 45.5% | 45.5% |  |
| Female | 54.5% | 54.5% | 0 |
| Year of birth | 1996.09 (0.48) | 1996.09 (0.48) | 0 |
| Completed higher education |  |  |  |
| Yes | 55.4% | 57.4% |  |
| No | 44.6% | 43.6% | 0.04 |
| Nationality |  |  |  |
| Russian | 79.1% | 79.1% |  |
| Non-Russian | 20.9% | 20.9% |  |

Table 5 presents group means, standard deviations, sample sizes, effect sizes (Cohen’s d), t-test values, and p-values for the comparison of changes in IDEA total scores across various groups, such as gender, living with parents, education, employment, relationship status, parenthood, nationality, substance use, mental health specialist consultation, and healthy lifestyle. Cohen’s d indicates effect size; t values are from independent samples t-tests; p values are two-tailed.

**Table 5**

Descriptive Statistics, Effect Sizes, and t-Test Results for Study Variables in Relation to Changes in IDEA Total Scores

| Variable | Group 1 (M, SD, n) | Group 2 (M, SD, n) | Cohen’s d | t | p |
| --- | --- | --- | --- | --- | --- |
| Gender | Male (-0.23, 3.69, 1011) | Female (-0.41, 3.69, 1211) | 0.05 | 1.18 | .24 |
| Life with parents | No (-0.35, 3.72, 1813) | Yes (-0.49, 3.66, 188) | 0.04 | 0.49 | .62 |
| Education | Not studying (-0.37, 3.67, 1940) | Studying (-0.06, 3.86, 282) | -0.08 | -1.27 | .20 |
| Completed higher education | Yes (-0.04, 3.62, 1386) | No (-0.79, 3.78, 812) | -0.17 | -4.51 | <.001 |
| Employment | Does not work (-0.72, 3.77, 528) | Works (-0.21, 3.66, 1694) | -0.13 | -2.72 | .007 |
| Relationships | Not involved (0.24, 3.66, 728) | In relationship (0.12, 3.69, 658) | 0.03 | 0.64 | .52 |
| Children | No children (0.004, 3.63, 1680) | At least one child (-1.37, 3.70, 542) | 0.37 | 7.54 | <.001 |
| Nationality | Not Russian (-0.29, 3.76, 460) | Russian (-0.34, 3.67, 1762) | 0.01 | 0.27 | .79 |
| Strong alcohol (weekly+) | Yes (-0.60, 3.56, 203) | No (-0.30, 3.70, 2019) | -0.08 | -1.13 | .26 |
| Smokes ≥1 pack/week | Yes (-0.47, 3.76, 320) | No (-0.30, 3.69, 1902) | 0.04 | -0.73 | .47 |
| Ever seen mental health specialist | No (-0.48, 3.66, 1796) | Yes (0.31, 3.77, 426) | -0.21 | -3.95 | <.001 |
| Declares healthy lifestyle | Yes (-0.07, 3.68, 1254) | No (-0.62, 3.68, 795) | 0.15 | 3.27 | .001 |

Table 6 shows means, standard deviations, sample sizes, effect sizes (Cohen’s d), and ANOVA results for the comparison of changes in IDEA total scores across age groups. F values are from one-way ANOVA.

**Table 6**

Descriptive Statistics, Effect Size, and ANOVA Results for Age Groups in Relation to Changes in IDEA Total Scores

| Variable | Group (M, SD, n) | Cohen’s d | F (df) | p |
| --- | --- | --- | --- | --- |
| Age | 1994–1995 (-0.03, 3.72, 143) |  | F(2, n) = 1.4 | .25 |
|  | 1996 (-0.40, 3.71, 1713) |  |  |  |
|  | 1997–1998 (-0.11, 3.59, 366) |  |  |  |

Table 7 presents Pearson correlation coefficients and effect sizes (Cohen’s d) for the associations between changes in IDEA total scores and other continuous study variables, including material well-being, health status, stress frequency, anxiety about the future, and change in anxiety. Asterisks indicate statistical significance (*p < .05, **p < .01).

**Table 7**

Correlations between changes in IDEA total scores and other study variables, with effect sizes

| Variable | Correlation Coefficient | Cohen’s d |
| --- | --- | --- |
| Material well-being | 0.13** (n=2119) | 0.26 |
| Health status | -0.12* (n=2222) | -0.25 |
| Stress frequency | -0.03 (n=2194) | -0.05 |
| Anxiety about the future | -0.02 (n=2222) | -0.04 |
| Change in anxiety | -0.08** (n=2010) | -0.17 |

Table 8 summarizes the results of a multiple regression analysis predicting changes in IDEA scores from 2021 to 2022. The table includes unstandardized regression coefficients (b), 95% confidence intervals (CI), semi-partial correlation squared (sr²), and p-values for each predictor. The model’s R², F statistic, and overall significance are also reported.

**Table 8**

Results of Multiple Regression Analysis Predicting Change in IDEA Scores from 2021 to 2022

| Predictor | b | 95% CI | sr² | 95% CI | p |
| --- | --- | --- | --- | --- | --- |
| Intercept | -2.02 | [-2.64, -1.39] |  |  | < .01 |
| Children (has at least one child) | -1.24 | [-1.60, -0.89] | .02 | [.01, .03] | < .01 |
| Psychologists (seen a mental health specialist) | 0.67 | [0.28, 1.06] | .01 | [-.00, .01] | < .01 |
| Subjective evaluation of material well-being | 0.48 | [0.32, 0.63] | .02 | [.01, .03] | < .01 |
